# Diagnostic value of C-reactive protein to rule out infectious complications after major abdominal surgery: a systematic review and meta-analysis

**DOI:** 10.1007/s00384-015-2205-y

**Published:** 2015-05-03

**Authors:** Sarah L. Gans, Jasper J. Atema, Susan van Dieren, Bas Groot Koerkamp, Marja A. Boermeester

**Affiliations:** Department of Surgery (G4-133), Academic Medical Center, 1105 AZ Amsterdam, The Netherlands; Clinical Research Unit, Academic Medical Center, Amsterdam, The Netherlands; Department of Anesthesiology, Academic Medical Center, Amsterdam, The Netherlands; Department of Surgery, Erasmus MC, Rotterdam, The Netherlands

**Keywords:** Abdominal surgery, C-reactive protein, Diagnostic accuracy, Postoperative infectious complications

## Abstract

**Purpose:**

Infectious complications occur frequently after major abdominal surgery and have a major influence on patient outcome and hospital costs. A marker that can rule out postoperative infectious complications (PICs) could aid patient selection for safe and early hospital discharge. C-reactive protein (CRP) is a widely available, fast, and cheap marker that might be of value in detecting PIC. Present meta-analysis evaluates the diagnostic value of CRP to rule out PIC following major abdominal surgery, aiding patient selection for early discharge.

**Methods:**

A systematic literature search of Medline, PubMed, and Cochrane was performed identifying all prospective studies evaluating the diagnostic value of CRP after abdominal surgery. Meta-analysis was performed according to the PRISMA statement.

**Results:**

Twenty-two studies were included for qualitative analysis of which 16 studies were eligible for meta-analysis, representing 2215 patients. Most studies analyzed the value of CRP in colorectal surgery (eight studies). The pooled negative predictive value (NPV) improved each day after surgery up to 90 % at postoperative day (POD) 3 for a pooled CRP cutoff of 159 mg/L (range 92–200). Maximum predictive values for PICs were reached on POD 5 for a pooled CRP cutoff of 114 mg/L (range 48–150): a pooled sensitivity of 86 % (95 % confidence interval (CI) 79–91 %), specificity of 86 % (95 % CI 75–92 %), and a positive predictive value of 64 % (95 % CI 49–77 %). The pooled sensitivity and specificity were significantly higher on POD 5 than on other PODs (*p* < 0.001).

**Conclusion:**

Infectious complications after major abdominal surgery are very unlikely in patients with a CRP below 159 mg/L on POD 3. This can aid patient selection for safe and early hospital discharge and prevent overuse of imaging.

**Electronic supplementary material:**

The online version of this article (doi:10.1007/s00384-015-2205-y) contains supplementary material, which is available to authorized users.

## Introduction

Postoperative infectious complications (PICs) occur frequently after major abdominal surgery. PICs have a major influence on patient outcomes and hospital costs [1–8]. A timely diagnosis of infectious complications is associated with a lower morbidity and mortality rate [7, 9]. However, early clinical features of postoperative infections are often nonspecific and difficult to distinguish from the normal postoperative inflammatory response related to surgical trauma [9]. The median time to diagnosis of infectious complications has been reported up to 12 days after surgery, with commonly several days of delay in retrospect [8].

A biological marker that can predict infectious complications before clinical signs and symptoms develop could be of clinical value. The value of such a marker is two-sided; it could identify patients with a high probability of infectious complications for early additional investigations, such as an abdominal CT scan, or it could identify patients with a low probability of infectious complications.

C-reactive protein (CRP) is a biological marker that might be of value in detecting infectious complications. CRP is a widely available, fast, and cheap marker. CRP levels are known to increase in the postoperative period, because of surgical tissue damage. CRP levels tend to normalize rapidly in patients with an uncomplicated postoperative course due to its short plasma half-life of 19 h [10, 11].

CRP has been extensively studied for its value in predicting PIC after major abdominal surgery [12–16]. Several studies have concluded that CRP is a useful predictor of PIC, but low positive predictive values have been reported [7, 16–23], making CRP a suboptimal marker for ruling in of an infectious complication. A recent meta-analysis of CRP after gastroesophageal cancer surgery confirms that CRP values are insufficient to predict postoperative inflammatory conditions [24].

The value of CRP to rule out the presence of infectious complications has not yet been studied. In an era of minimal invasive surgery and enhanced recovery programs, patients are often discharged early, possibly before clinical signs of deterioration have become evident. A marker that accurately predicts the absence of postoperative complications could aid patient selection for safe and early hospital discharge and prevent overuse of imaging.

The present systematic review and meta-analysis aims to determine the value of CRP to rule out the presence of infectious complications allowing for safe and early discharge of patients after major abdominal surgery.

## Material and methods

### Search strategy

Embase, PubMed, and the Cochrane library were searched up to the 26 of January 2014. The search strategy consisted of the MeSH terms and free text words indexed for CRP and major abdominal surgery. The detailed search strategy is available in Appendix A. This review was performed according to the Preferred Reporting Items for Systematic Reviews and Meta-Analyses (PRISMA) statement.

### Study selection

Inclusion and exclusion criteria were set before the search. Articles were considered eligible if the diagnostic accuracy of CRP for PIC following abdominal or gastrointestinal surgery was assessed in a prospective study design. If the following criteria were all met, articles were included: (1) CRP was evaluated in the postoperative setting, (2) CRP was evaluated after major abdominal or gastrointestinal surgical procedures (including pancreatic, colorectal, hepatobiliary, esophageal, and gastric surgery), (3) outcome of interest was the association between CRP and PIC, and (4) the study design was prospective. Designs other than prospective design were excluded to minimize the risk of bias. Studies presenting insufficient data for extracting 2 × 2 contingency tables of CRP versus PIC were also excluded. Original articles in the English, French, German, Dutch, or Spanish language were considered for inclusion.

**Fig. 1:**
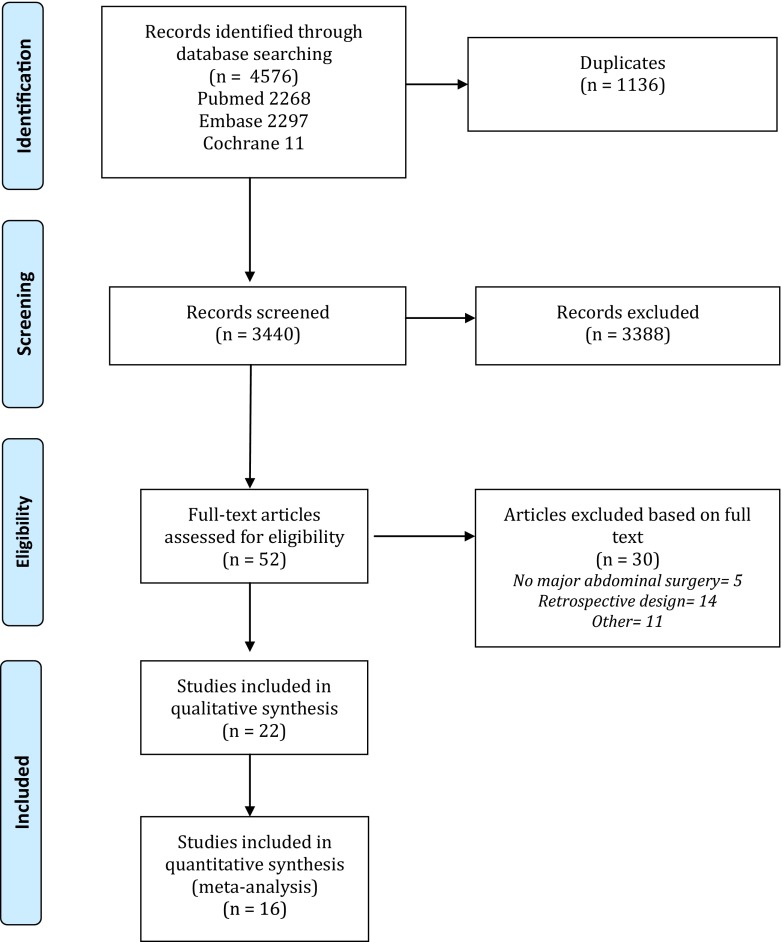
PRISMA flow chart

Two independent reviewers (SLG and JJA) screened the titles and abstracts of all papers identified by the search for eligibility. The full text was obtained of potentially eligible papers for further evaluation. Reference lists of key articles and reviews were manually searched to identify additional articles. In case of disagreement, consensus was reached through discussion. The inclusion and exclusion of articles were recorded in a PRISMA flow chart (Fig. 1). Two reviewers independently extracted data from the included studies using a standardized form (SLG, JJA).

### Test outcome

CRP levels were compared for patients with and without PIC. PIC was defined as reported in the studies. If provided, outcomes were registered for in-hospital stay and 30-day period. CRP data were recorded whenever mentioned in text, graphs, or figures of the article. Data regarding measures of diagnostic accuracy such as sensitivity, specificity, positive predictive value, negative predictive value, and area under the receiver-operating curve (AUC) were recorded as reported in the included articles. The cutoff value of CRP with presumed highest discriminatory value was recorded.

### Reference standard

The outcome of interest was PIC. PICs were counted per event and defined as reported in the individual studies. The true disease status or reference standard, i.e., whether patients actually developed a PIC, could be determined in multiple ways. Follow-up, surgery, and radiological imaging were all accepted as reference standard for the diagnosis of PIC. Duration of clinical follow-up was recorded.

### Study design, patient characteristics, and quality

The following data were extracted from included studies: study period; department of the first author; inclusion period; study design; country of origin; and patient characteristics such as number of included patients, the mean or median age (and range), male to female ratio, time of follow-up, and the number of true positives (TP), false positives (FP), false negatives (FN), and true negatives (TN). The methodological quality of the studies was assessed using the Quality Assessment of Diagnostic Accuracy Studies 2 (QUADAS-2) tool [25, 26]. Two by two contingency tables were extracted or reconstructed for CRP versus PIC for every included study.

### Meta-analysis

Meta-analysis was performed with studies that provided sufficient quantitative data to calculate a contingency table for a specified cutoff value of CRP at a specified postoperative day (POD). A nonlinear mixed model was used to obtain summary estimates of sensitivity and specificity with 95 % confidence intervals (CIs) [27]. To compare sensitivity and specificity for each POD, we used the Wald test for unpaired data. Pooled likelihood ratios (LRs), positive predictive values (PPVs), and negative predictive values (NPVs) were calculated for each POD using the pooled sensitivity and specificity. The geometric mean with 95 % CI was used to calculate the pooled CRP value per POD to compare patients with and without PIC.

The pooled cutoff value was calculated using the cutoff values provided in the individual studies per POD weighted by their sample size. The pooled area under the curve was calculated using the individual AUCs weighted by their sample size. The pooled incidence of PIC was calculated using the incidence of the individual studies weighted by their sample size. The pooled incidence was not necessarily the same for each POD, because for each POD, different studies were available for pooled analyses.

The pretest probability of developing a PIC can be thought of as the probability that a patient will develop a PIC based on bedside evaluation. The posttest probability is the probability that a patient will develop a PIC based on both bedside evaluation and the CRP value. The posttest odds were calculated by multiplying the pretest odds with the positive and negative LR. Posttest probabilities for a high and a low CRPs were calculated and presented in a graph for pretest probabilities across the range of 0 to 100 %. The underlying assumption of this graph is that the positive and negative LRs were constant across all pretest probabilities. Using the pooled incidence, representing an average patient, as the pretest probability resulted in posttest probabilities for a positive and negative index test. This incidence can differ across PODs, depending on the studies available for pooled analysis for each POD.

All statistical analyses were performed using SPSS (version 20.0, IBM, Armonk, New York, USA) and SAS (version 9.3, SAS institute Inc., Cary North Carolina). *P* values of <0.05 were considered to indicate statistical significance.

## Results

The search identified 3440 articles after excluding duplicate articles. Articles not meeting the inclusion criteria based on assessment of the title and abstract (3388) were excluded. Full text of the potentially eligible 52 articles was retrieved for detailed examination. Inclusion criteria were not met in 30 studies. Most of the excluded studies either had a retrospective design or assessed the value of CRP in settings other than major abdominal surgery [3, 10, 14, 17–19, 21, 28–38]. The remaining 22 studies were included for qualitative analysis of which 16 studies could also be used for meta-analysis (Fig. 1).

### Study and patient characteristics

The included articles were published between 1997 and 2013 (Table 1). CRP levels of 2215 patients were examined for their value in predicting PIC in patients undergoing major abdominal surgery. Postoperative follow-up duration varied from 7 up to 60 days postoperative. Further study characteristics are summarized in Table 1. Most studies analyzed the value of CRP in colorectal surgery (eight studies), gastrectomy (three studies), and esophagectomy (three studies).

**Table 1:** Characteristics of included studies

Reference	Inclusion period	No. of participants	Type of surgery	Follow-up duration	Age	Female sex (%)	Primary outcomes
Aguilar-Nascimento [39], Brazil	2004–2005	32	Major gastrointestinal surgery with at least one anastomosis	NS	49 (range 18–72)	14 (46 %)	Infectious morbidity
Albanopoulos [40], Greece	2008–2011	177	Laparoscopic sleeve gastrectomy	30 days	38.1 (range 18–61)	102 (58 %)	Postoperative complications within 30 days
Dutta [14], UK	2005–2009	136	Esophagogastric cancer resections	7 days	(Age <65, 65–75, >75)	37 (27 %)	Postoperative complications
					No complications 37/25/7		
					Infectious complications 26/21/7		
					Noninfectious complications 6/6/1		
Garcia-Granero [41], Spain	2008–2010	205	Elective colorectal surgery with primary intestinal anastomosis	60 days after discharge	63.3 SD (15.5)	93 (45, 4 %)	Complications
Guirao [19], Spain	2007–2009	208	Open or laparoscopic colorectal surgery	30 days	68.3 SD (11.4)	82 (39.4 %)	Organ space infection
Lagoutte^a^ [42], France	2010–2011	100	Elective colonic or rectal surgery with immediate restoration of intestinal continuity	30 days after surgery	64 (range 20–87)	42 (42 %)	Complications
Mackay [2], UK	2003–2006	150	Elective colorectal resection	30 days	72 (IQR 63–79)	78 (52 %)	Infective complications
Matsuda [25], Japan	2006–2007	41	Elective colorectal surgery	30 days	Uninfected 69.5 ± 2.0	Uninfected 15 (52 %)	Postoperative infections
					Infected 69.7 ± 2.7	Infected 1 (8.3 %)	
Matthiessen^a^ [1], Sweden	2002–2003	33	Anterior resection for rectal carcinoma	NS	68 (range 38–80)	11 (33 %)	Anastomotic leakage
Oberhofer [43], Croatia	2009	79	Elective colorectal surgery	NS	Complications 65.7	29 (37 %)	Complications
					No complications 79.8		
Ortega–Deballon [30], France	2007–2008	133	Elective colorectal surgery	6 weeks	65 ± 16	48 (36 %)	Septic complications (including leaks, wound infections, central line infection, urinary tract infection, pneumonia)
Platt [13], UK	1997–2007	454	Curative resection for colorectal cancer	7 days	(Age <65, 65–75, >75)	275 (61 %)	Complications (infective and noninfective)
					No complications 120/99/115		
					Infective complications 30/41/33		
					Noninfective complications 1/8/7		
Ramanathan^a^ [44], UK	NS	357	Curative surgery for colorectal cancer	30 days	(<65/65–74/>74 years)	169 (47 %)	Infective complications
					99/113/145 (28/32/40 %)		
Reith^a^ [37] Germany	NS	70	Aortal surgery (*n* = 35)	10 days	NS	NS	Complications
			Colorectal surgery (*n* = 35)				
Scepanovic [45], Serbia and Montenegro	2010–2012	156	Elective abdominal surgery with primary anastomosis	30 days	65 (28–86)	67 (42.9 %)	Postoperative complications (infectious and noninfectious)
Shimizu[46], Japan	1997–1999	112	Gastrointestinal surgery	4 weeks	Noninfected 60 ± 2	65 (58 %)	Infectious complications
					Minor infected 68 ± 2		
					Severely infected 68 ± 4		
Siassi^a^ [47], Germany	2000–2001	172	Major elective surgery for malignant disease of the gastrointestinal tract	NS	62.3 (25–83)	116 (67 %)	Postoperative complications
Takakura [48], Japan	2010–2011	114	Colonic surgery	NS	Noncomplicated 65 (32–92)	Noncomplicated 33 (85 %)	SSI (superficial and deep)
					Complicated 61 (42–82) median, range	Complicated 6 (15 %)	
Van Genderen [4], The Netherlands	2007–2008	63	Elective esophagectomy with gastric tube reconstruction	10 days after surgery	61 ± 8.9	18 (29 %)	Complications
Veeramootoo^a^ [49], UK	2004–2006	50	Minimally invasive esophagectomy	NS	67 (47–81)	5 (10 %)	Complications
Welsch [50], Germany	2002–2005	688	Pancreatic resections with pancreaticojejunostomy for neoplasms and chronic pancreatitis	12 days	NS	NS	Inflammatory postoperative complications
Welsch [5], Germany	2001–2005	96	Rectal resections with sphincter preserving primary anastomosis	12 days	Complicated: 65.0 (59.7–75.5)	28 (29 %)	Infectious postoperative complications
					No complications 66.8 (61.1–71.8)		

*NS* not specified

^a^Studies not included in meta-analysis

### Quality of included studies/risk of bias

**Fig. 2:**
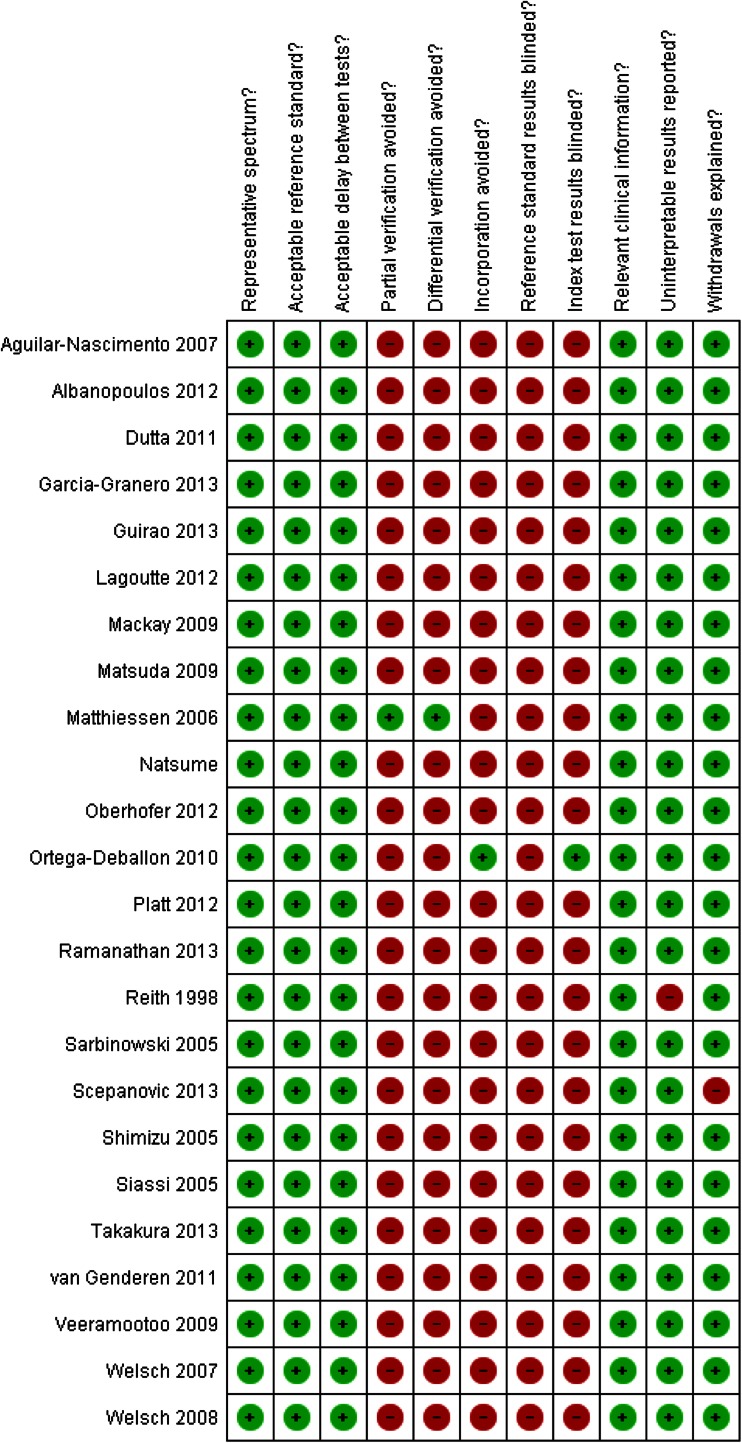
Methodological quality summary of the included studies

**Fig. 3:**
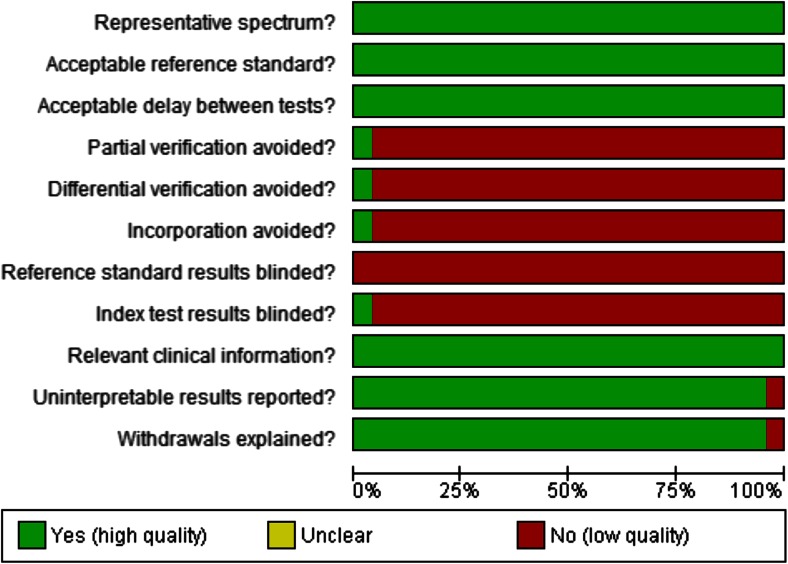
Methodological quality across studies

The quality of the included studies was fairly good (Figs. 2 and 3). All studies had a representative spectrum of patients and used an acceptable reference standard. The time between index and reference test was acceptable in all studies. The preferred reference standard differed across studies. In most studies, only patients with elevated values of inflammatory markers or a clinical suspicion of complications underwent imaging as diagnostic reference standard (*partial verification*). The preferred reference test differed between patients with a positive index test (e.g., patients with elevated inflammatory markers) and negative index test. The preferred reference standard in patients with a positive index test was diagnostic imaging (predominantly computed tomography or conventional radiography with water-soluble contrast), whereas in patients with a negative index test, clinical follow-up was the reference standard (*differential verification*). Only one study avoided partial and differential verification by performing imaging in all patients [1]. In only one study, incorporation of the index test in the reference test was avoided [33]. None of the studies blinded the outcome assessors for the reference standard. In only one study, the index test results were blinded [33]. All studies provided information on uninterpretable results except for one study [22]. One study failed to provide information on withdrawals [51].

Overall, the risk of bias in the studies is low due to the nature of the index test (CRP). The outcome of the index test was independent of the reference standard. In all studies, CRP measurement was performed in a standardized manner for study purposes and independent of clinical suspicion of infection, nor was clinical suspicion of infection documented.

### Predictive value of CRP for infectious complications

The incidence of PIC ranged from 5 to 60 % across studies. The average incidence was 27 % (95 % CI; 26–29 %).

The cutoff level for CRP, which was used to calculate sensitivity, specificity, NPV, and PPV, varied across studies from 48 to 200 mg/L. In most studies, CRP levels were significantly higher in patients with infectious complications compared to patients without complications. This difference increased each POD (Tables 2 and S2).

**Table 2:** Mean CRP levels per POD in relation to complications

POD	Number of studies (*n* = patients)	Mean CRP level (95 % CI)	Mean CRP level (95 % CI)
		Complicated (infectious)	Uncomplicated
1	5 (*n* = 593)	122 (52–288)	67 (36–123)
2	5 (*n* = 641)	195 (91–420)	146 (65–329)
3	5 (*n* = 593)	190 (125–289)	98 (50–195)
4	2 (*n* = 338)	170 (165–174)	95 (78–116)
5	5 (*n* = 663)	188 (71–497)	62 (28–139)

**Fig. 4:**
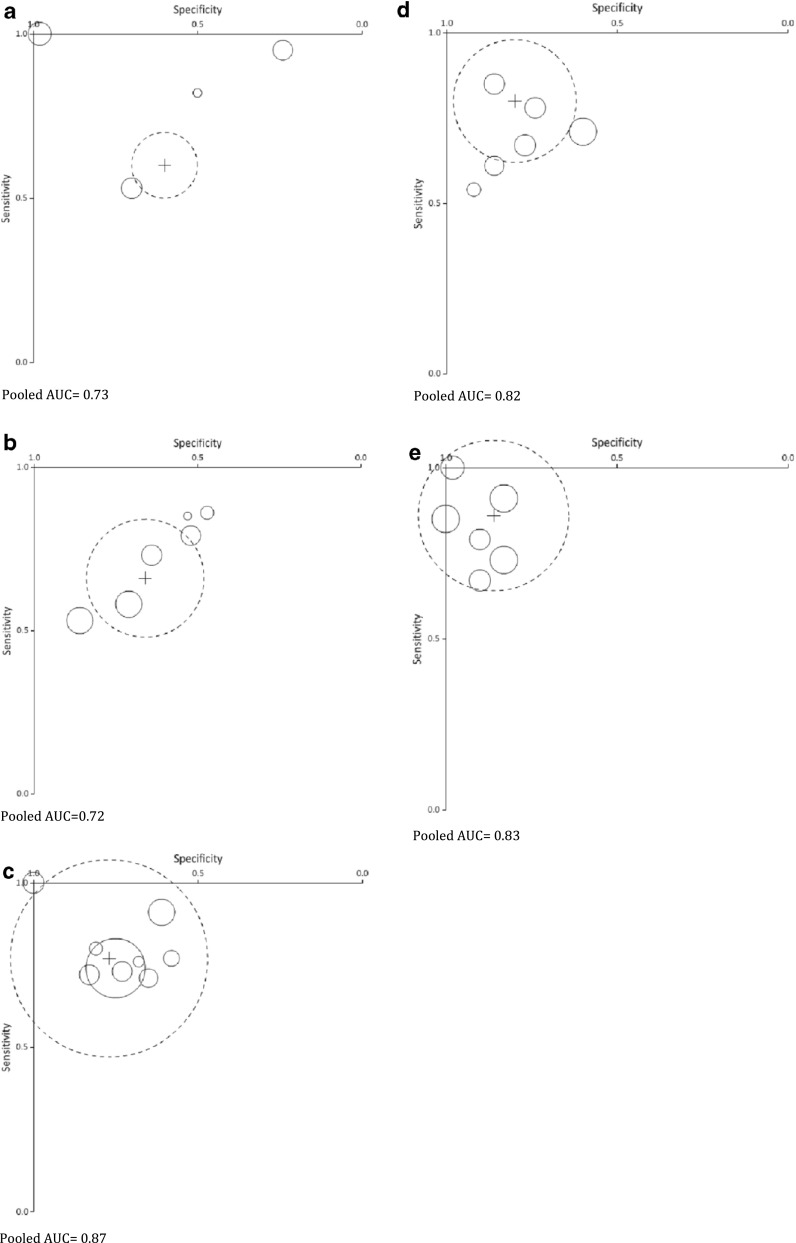
Bubble plot of sensitivity and specificity of the individual studies including the pooled values (*circle with dashed line* is the pooled AUC circle with *black lines* representing the individual studies weighted by their sample size). **a** On postoperative day 1. Pooled AUC = 0.73. **b** On postoperative day 2. Pooled AUC = 0.72. **c** On postoperative day 3. Pooled AUC = 0.87. **d** On postoperative day 4. Pooled AUC = 0.82. **e** On postoperative day 5. Pooled AUC = 0.83

Four studies provided sufficient data for meta-analysis on POD 1, nine studies provided data for POD 3, and six studies provided data for POD 2, POD 4, and POD 5. Sensitivity, specificity, PPV, and NPV from the studies are provided in Table S1 (Appendix). Values of sensitivity and specificity of the individual studies are plotted for each POD in Fig. 4a–e. Sensitivity and specificity increased up to POD 3. The pooled AUC ranged from 0.72 on POD 2 to 0.87 on POD 3 and 0.83 on POD 5. Up to POD 2, the pooled cutoff value of CRP increased. From POD 3 onward, the pooled cutoff value decreased (Table 3). The pooled cutoff value for the CRP was 190 mg/L (range 140–240) on POD 2, 159 mg/L (range 92–200) on POD 3, and 114 mg/L (range 48–150) on POD 5.

**Table 3:** Pooled diagnostic accuracy of included studies

POD	Number of studies (*n* = patients)	Pooled incidence PIC (%)	Pooled AUC	Pooled CRP cutoff (range)	Pooled sensitivity (95 % CI)	Pooled specificity (95 % CI)	Pooled PPV (95 % CI)	Pooled NPV (95 % CI)	Pooled LR+ (95 % CI)	Pooled LR− (95 % CI)
1	4 (*n* = 546)	18	0.73	157 (109–187)	60 % (47–71 %)	60 % (43–75 %)	41 % (27–56 %)	82 % (68–90 %)	1.48 (0.66–2.30)	0.67 (0.33–1.02)
2	6 (*n* = 881)^a^	24	0.72	190 (140–240)	66 % (54–76 %)	66 % (50–79 %)	45 % (31–60 %)	84 % (72–91 %)	1.95 (0.87–3.03)	0.51 (0.27–0.76)
3	9 (*n* = 1567)	32	0.87	159 (92–200)	77 % (68–84 %)	77 % (64–87 %)	57 % (43–71 %)	90 % (81–95 %)	3.41 (1.43–5.39)	0.29 (0.16–0.43)
4	6 (*n* = 894)	33	0.82	132 (101–180)	80 % (71–86 %)	80 % (67–88 %)	60 % (45–73 %)	91 % (83–95 %)	3.93 (1.58–6.28)	0.26 (0.13–0.38)
5	6 (*n* = 1104)^a^	17	0.83	114 (48–150)	86 % (79–91 %)	86 % (75–92 %)	64 % (49–77 %)	92 % (85–96 %)	6.07 (2.26–9.89)	0.17 (0.09–0.25)

*POD* postoperative day, *AUC* area under the receiver operating curve, *PPV* positive predictive value, *NPV* negative predictive value, *LR+* positive likelihood ratio, *LR−* negative likelihood ratio

^a^One study analyzed patients in two groups, laparoscopic vs open. Patients of the two groups were included separately in the analysis (as reported in the study)

Pooled diagnostic accuracy variables are listed in Table 3. The pooled sensitivity and specificity increased per POD. The lowest pooled sensitivity and specificity were reached on POD 1 (respectively, 60 %; 95 % CI (47–71 %) and 60 %; 95 % CI (43–75 %)). Pooled sensitivity and specificity were highest on POD 5, 86 % (79–91 %) and 86 % (75–92 %), and were significantly higher on POD 5 compared to all other PODs (*p* < 0.001). Using the pooled cutoff values would lead to 23 % of missed cases (1—sensitivity) of PIC on POD 3, 20 % on POD 4, and 14 % on POD 5.

The pooled NPV increased each day after surgery at a decreasing cutoff of the CRP values (Table 3). The NPV ranged from 82 % (95 % CI; 68–90 %) on POD 1 to 92 % (95 %CI; 85-96 %) on POD 5. The pooled PPV was low ranging from 41 % (95 % CI; 27–56 %) on POD 1 to 64 % (95 % CI; 49–77 %) on POD 5.

The negative likelihood ratio (LR−) decreased each POD. The highest LR− was 0.67 (95 % CI; 0.33–1.02) on POD 1, and the lowest LR− was 0.17 (0.09–0.25) on POD 5. The positive likelihood ratio (LR+) of CRP increased each POD. The lowest pooled LR+ was 1.48 (95 % CI; 0.66–2.30) on POD 1, and the highest LR+ was 6.07 (95 % CI; 2.26–9.89) on POD 5.

**Fig. 5:**
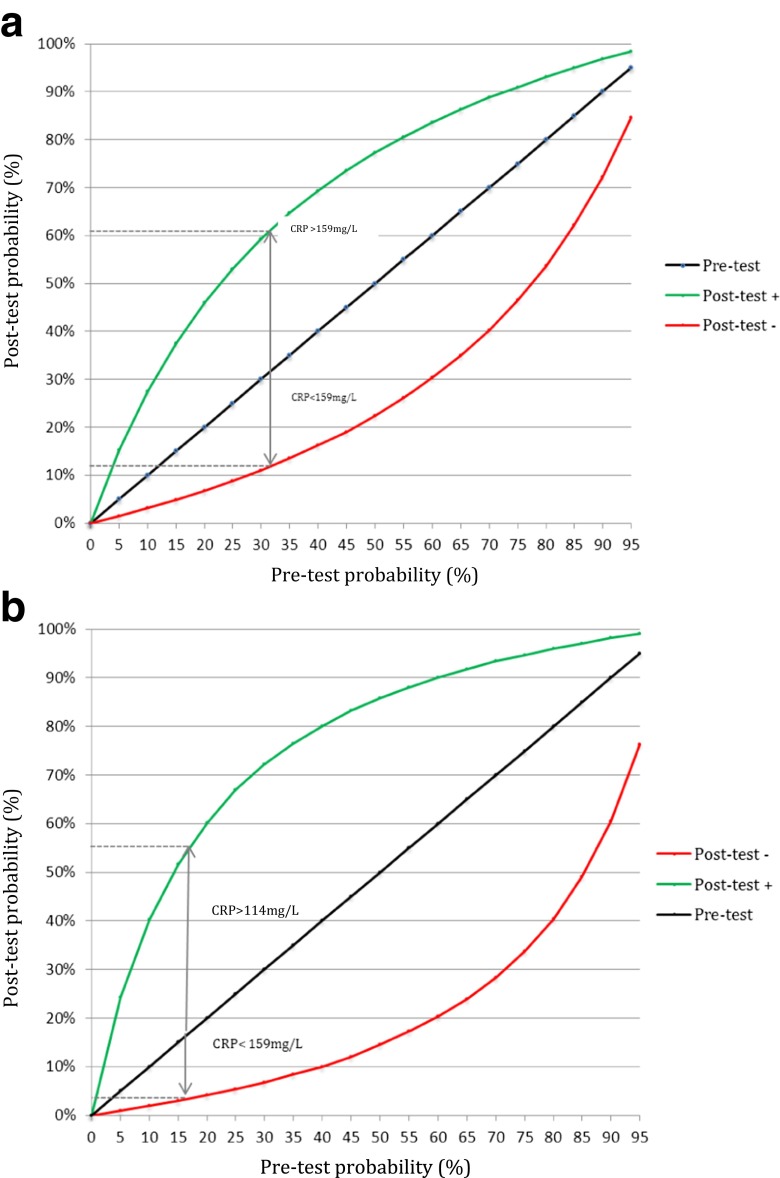
**a** The (posttest) probability of a PIC is presented for a patient with a high CRP (*green line*) and a low CRP (*red line*) on POD 3 (**a**) and POD 5 (**b**). The cutoff value between a low and a high CRP was 159 mg/L on POD 3 and 114 mg/L on POD 5. The *arrows* show that the posttest probability of a PIC for an average patient (incidence of PIC 32 %) with a high CRP on POD 3 was 61 versus 12 % in an average patient with a low CRP. The *length of the arrows* represents the absolute change in probability of a PIC in case of a high or low CRP. On POD 5, an average patient (incidence of PIC 16 %) with a high CRP had a posttest probability of 55 versus 3 % in an average patient with a low CRP. The *black diagonal line* at 45° with the *x*-axis represents the line of a hypothetical noninformative test in which the pretest and posttest probabilities are equal. The posttest probability of a PIC can be read from the two panels for any pretest probability (i.e., based on bedside evaluation) and CRP value. Pretest probability (incidence) = 0.32, posttest + probability = CRP >159 mg/L = 0.61 and posttest – probability = CRP <159 mg/L = 0.12. **b** Posttest probability as a function of pretest probability for the positive and negative likelihood ratio on POD 5

Figure 5 presents the (posttest) probability of a PIC for a patient with a high CRP (green line) and a low CRP (red line) on POD 3 (Fig. 5a) and POD 5 (Fig. 5b). The cutoff value between a low and a high CRP was 159 mg/L on POD 3 and 114 mg/L on POD 5. The posttest probability of a PIC in an average patient with a high CRP on POD 3 was 61 versus 12 % in an average patient with a low CRP. On POD 5, an average patient with a high CRP had a posttest probability of 55 versus 3 % in an average patient with a low CRP.

## Discussion

The objective of this meta-analysis was to evaluate the value of CRP to rule out PICs after abdominal and gastrointestinal surgery. In the era of fast-track surgery where patients are discharged early after surgery and mostly within the first five PODs, there is a need for a reliable, inexpensive, and widely available marker that permits safe and early discharge of patients. A marker reliable enough to rule out the presence of infectious complications would have a high NPV and a low negative LR. The NPV of CRP is higher than 90 % from day 3 onward. This suggests that patients with a CRP below 159 mg/L on POD 3 have a low probability of developing a PIC and could safely be discharged early.

A recent meta-analysis that focused on the diagnostic value for the presence of anastomotic leakage also found a high NPV justifying early discharge of patients after colorectal surgery [52]. However, this meta-analysis is limited by the methodological design of the included studies. The majority of included studies have a retrospective design resulting in significant heterogeneity. A retrospective study design leads to selective measurement of CRP in patients who are clinically suspected of having infectious complications (incorporation bias), which may lead to an overestimation of diagnostic accuracy.

In many European centers, CRP is used in daily practice in combination with clinical judgment based on history and physical examination. Combining CRP with clinical judgment might increase the (negative) predictive value of CRP even more. Bedside evaluation during the postoperative course broadly classifies patients into three categories. Firstly, there is a group of patients without a clinical suspicion of infectious complications and, thus, a very low pretest probability. In these patients, even an elevated CRP value on POD 5 may not increase the posttest probability enough to warrant a change in management (e.g., imaging or antibiotics). CRP is of limited value for these patients. The second group includes patients in whom the suspicion of infectious complications based on clinical evaluation is very high. In these patients, the (posttest) probability will remain sufficiently high, even for a low CRP value, to justify a change in management. CRP again has limited value in decision making for these patients. Finally, predominantly in patients with an intermediate pretest probability of infectious complications, CRP values are most likely to determine the need for a change in management. For high CRP values, the (posttest) probability of an infectious complication might be sufficiently high to justify a change in management, while a low CRP value might justify no change in management. Figure 5 can be used as a decision aid, in which the physician still needs to determine the (posttest) probability of a PIC above which he or she feels that a change in management is warranted.

Studies have also demonstrated that CRP values initially increase postoperatively and then tend to normalize in patients without infectious complications around POD 3 [52]. Other studies have confirmed this suggestion [1, 16, 23, 28, 53, 54]. The results of the present meta-analysis confirm these results demonstrating that average values of CRP differ between patients with and without infectious complications. These findings suggest that prolonged elevated CRP values are predictive of infectious complications. CRP might be clinically useful to aid selection of patients for additional imaging. In this review, the highest PPV of CRP was 64 % on POD 5. This PPV would lead to a FP diagnosis in 36 % of patients, resulting in unnecessary additional imaging in these patients.

Diagnostic test research can be subject to several limitations. Firstly, knowledge of the CRP value might influence the interpretation of the reference test. For example, the same intraabdominal fluid collection on a CT scan may be classified as abscess if the CRP is high, but as ascites if the CRP is low. Only in one study, blinding for the outcomes of CRP value was used when determining the presence or absence of PIC [33]. Another difficulty in diagnostic test research is the heterogeneity across studies for the selected optimal CRP cutoff value. The optimal cutoff value is selected by the authors of the individual studies at the level at which they feel patients with and without infectious complications are best distinguished. At a higher cutoff value for the CRP, the sensitivity is higher, but the specificity is lower. To prevent bias, ideally, the analysis should have been performed using the same cutoff value of the CRP in each study. Unfortunately, insufficient data was reported to use a single optimal cutoff value of the CRP. Pooling study results even with small differences in the cutoff value have inevitably biased the results. Diagnostic test results can also be influenced by variation in timing of the reference test: for example, whether a CT scan to detect an intraabdominal abscess was performed early or late during follow-up. Another limitation is the difference in the definition of infectious complications between studies. This might influence the incidence of infectious complications. We aimed to minimize the risk of bias by including studies that used radiological and/or clinical evidence to define infectious complications. Finally, the pooled analysis included patients who underwent different types of surgery. A bias may have been introduced because the prognostic value of CRP may depend on the type of surgery. It has been demonstrated that the postoperative increase in CRP is dependent on the extent of operative trauma. Different types of surgery could lead to different absolute values of CRP. Nevertheless, this discrepancy between types of abdominal surgery can only exist in the first 2 days due to the short half-life of CRP (19 h). The trend where CRP values tend to normalize in patients without infectious complications around POD 3 has been demonstrated for various types of surgery [1, 16, 23, 28, 53, 54]. The major advantage of combining different types of abdominal surgery is that a much larger sample size was reached resulting in more precise estimates.

This meta-analysis evaluated the value of CRP as a predictor to rule out infectious complications. However, when CRP is combined with bedside clinical evaluation, as used in daily practice, the NPV of CRP might further increase as illustrated in Fig. 5. In the literature, no studies were found that assessed the added value of CRP on top of bedside judgment. Future studies should aim to determine this added value of CRP. These studies should also evaluate the diagnostic value of the change in CRP in the postoperative period instead of focusing on the absolute value. The change in CRP (e.g., between POD 2 and 5) may be a stronger predictor than the absolute CRP value on POD 5. Also, the actual benefit of early detection and management is assumed but needs to be determined more definitively. In conclusion, CRP values seem clinically useful to aid patient selection for safe and early hospital discharge and prevent overuse of imaging.

## Electronic supplementary material

ESM 1 (124 kb)

## References

[1] Matthiessen P, Henriksson M, Hallböök O, Grunditz E, Norén B, Arbman G (2008). Increase of serum C-reactive protein is an early indicator of subsequent symptomatic anastomotic leakage after anterior resection. Color Dis.

[2] MacKay GJ, Molloy RG, O’Dwyer PJ (2011). C-reactive protein as a predictor of postoperative infective complications following elective colorectal resection. Color Dis.

[3] Warschkow R, Tarantino I, Folie P, Beutner U, Schmied BM, Bisang P (2012). C-reactive protein 2 days after laparoscopic gastric bypass surgery reliably indicates leaks and moderately predicts morbidity. J Gastrointest Surg.

[4] Van Genderen ME, Lima A, de Geus H, Klijn E, Wijnhoven B, Gommers D (2011). Serum C-reactive protein as a predictor of morbidity and mortality in intensive care unit patients after esophagectomy. Ann Thorac Surg Elsevier Inc.

[5] Welsch T, Müller SA, Ulrich A, Kischlat A, Hinz U, Kienle P (2007). C-reactive protein as early predictor for infectious postoperative complications in rectal surgery. Int J Color Dis.

[6] Warschkow R, Ukegjini K, Tarantino I, Steffen T, Müller SA, Schmied BM (2012). Diagnostic study and meta-analysis of C-reactive protein as a predictor of postoperative inflammatory complications after pancreatic surgery. J Hepatobiliary Pancreat Sci.

[7] Alves A (2005). Postoperative mortality and morbidity in French patients undergoing colorectal surgery. Arch Surg.

[8] Vonlanthen R, Slankamenac K, Breitenstein S, Puhan MA, Muller MK, Hahnloser D (2011). The impact of complications on costs of major surgical procedures: a cost analysis of 1200 patients. Ann Surg.

[9] Hyman N, Manchester T, Osler T (2007) Anastomotic leaks after intestinal anastomosis: it’s later than you think. Ann Surg, p 254–258 10.1097/01.sla.0000225083.27182.85 PMC1876987 17245179

[10] Bianchi R, Silva N, Natal M, Romero M (2004). Utility of base deficit, lactic acid, microalbuminuria, and C-reactive protein in the early detection of complications in the immediate postoperative evolution. Clin Biochem.

[11] Pepys MB, Hirschfield GM (2003). C-reactive protein: a critical update. J Clin Invest.

[12] Kørner H, Nielsen HJ, Søreide JA, Nedrebø BS, Søreide K, Knapp JC (2009). Diagnostic accuracy of C-reactive protein for intraabdominal infections after colorectal resections. J Gastrointest Surg.

[13] Rapport MM, Schwartz AE, Graf L (1957). C-reactive protein in patients following operation. Ann Surg.

[14] Woeste G, Müller C, Bechstein WO, Wullstein C (2010). Increased serum levels of C-reactive protein precede anastomotic leakage in colorectal surgery. World J Surg.

[15] Platt JJ, Ramanathan ML, Crosbie RA, Anderson JH, McKee RF, Horgan PG (2012). C-reactive protein as a predictor of postoperative infective complications after curative resection in patients with colorectal cancer. Ann Surg Oncol.

[16] Dutta S, Fullarton GM, Forshaw MJ, Horgan PG, McMillan DC (2011). Persistent elevation of C-reactive protein following esophagogastric cancer resection as a predictor of postoperative surgical site infectious complications. World J Surg.

[17] Meyer ZC, Schreinemakers JMJ, Mulder PGH, de Waal RAL, Ermens AAM, van der Laan L (2013). The role of C-reactive protein and the SOFA score as parameter for clinical decision making in surgical patients during the intensive care unit course. PLoS One.

[18] Mokart D, Merlin M, Sannini A, Brun JP, Delpero JR, Houvenaeghel G (2005). Procalcitonin, interleukin 6 and systemic inflammatory response syndrome (SIRS): early markers of postoperative sepsis after major surgery. Br J Anaesth.

[19] Noble F, Curtis NJ, Underwood TJ (2013). C-reactive protein 2 days after laparoscopic gastric bypass surgery reliably indicates leaks and moderately predicts morbidity. J Gastrointest Surg.

[20] Ortega-Deballon P, Facy O, Rat P (2012). Diagnostic accuracy of C-reactive protein and white blood cell counts in the early detection of infectious complications after colorectal surgery. Int J Color Dis.

[21] Takesue Y, Yokoyama T (1998). Prediction for the development of postoperative infections in the operation of esophageal cancer compared with gastric surgery. Hiroshima J Med Sci.

[22] Reith B, Mittelkötter U, Debus ES (2011). Procalcitonin in early detection of postoperative complications. Dig Surg.

[23] Molter GP, Soltész S, Kottke R, Wilhelm W, Biedler A, Silomon M (2003). Procalcitonin plasma concentrations and systemic inflammatory response following different types of surgery. Anaesthesist.

[24] Warschkow R, Tarantino I, Ukegjini K, Beutner U, Müller SA, Schmied BM (2012). Diagnostic study and meta-analysis of C-reactive protein as a predictor of postoperative inflammatory complications after gastroesophageal cancer surgery. Langenbecks. Arch Surg.

[25] Whiting P, Rutjes AWS, Reitsma JB, Bossuyt PMM, Kleijnen J (2003). The development of QUADAS : a tool for the quality assessment of studies of diagnostic accuracy included in systematic reviews. BMC Med Res Methodol.

[26] Reitsma JB, Leeflang MMG, Sterne JAC, Bossuyt PMM (2014) Research and reporting methods accuracy studies. Ann Intern Med (4)

[27] Leeflang MMG, Deeks JJ, Rutjes AWS, Reitsma JB, Bossuyt PMM (2012). Bivariate meta-analysis of predictive values of diagnostic tests can be an alternative to bivariate meta-analysis of sensitivity and specificity. J Clin Epidemiol Elsevier Inc.

[28] Almeida AB, Faria G, Moreira H, Pinto-de-Sousa J, Correia-da-Silva P, Maia JC (2012). Elevated serum C-reactive protein as a predictive factor for anastomotic leakage in colorectal surgery. Int J Surg Elsevier Ltd.

[29] Deitmar S, Anthoni C, Palmes D, Haier J, Senninger N, Brüwer M (2009). Are leukocytes and CRP early indicators for anastomotic leakage after esophageal resection?. Zentralbl Chir.

[30] Warschkow R, Steffen T, Beutner U, Müller SA, Schmied BM, Tarantino I (2012). Diagnostic accuracy of C-reactive protein and white blood cell counts in the early detection of inflammatory complications after open resection of colorectal cancer: a retrospective study of 1,187 patients. Int J Color Dis.

[31] Matsuda A, Matsutani T, Sasajima K, Furukawa K, Tajiri T, Tamura K et al (2009) Preoperative plasma adiponectin level is a risk factor for postoperative infection following colorectal cancer surgery. J Surg Res Elsevier Ltd 157(2):227–234 10.1016/j.jss.2008.09.007 19394964

[32] John B, Wijeyekoon S, Warnaar N (2010) Biochemical indicators of in-hospital complications following pancreatic surgery. Int Surg 95:215–220 21066999

[33] Ortega-Deballon P, Radais F, Facy O, d’Athis P, Masson D, Charles PE (2010). C-reactive protein is an early predictor of septic complications after elective colorectal surgery. World J Surg.

[34] Pedersen T, Roikjær O, Jess P (2012). Increased levels of C-reactive protein and leukocyte count are poor predictors of anastomotic leakage following laparoscopic colorectal resection. Dan Med J.

[35] Ravishankaran P, Shah AM, Bhat R (2011). Correlation of interleukin-6, serum lactate, and C-reactive protein to inflammation, complication, and outcome during the surgical course of patients with acute abdomen. J Interferon Cytokine Res.

[36] Reissfelder C, Rahbari NN, Koch M, Kofler B, Sutedja N, Elbers H (2011). Postoperative course and clinical significance of biochemical blood tests following hepatic resection. Br J Surg.

[37] Eriksson S, Olander B, Pira U, Granstrom L (1997) White blood cell count, leucocyte elastase activity and serum concentrations of interleukin-6 and C-reactive protein after open appendicectomy. Eur J Surg, p 123–127 9076439

[38] Yang M, Jeng L, Kao A (2002). C-reactive protein and gallium scintigraphy in patients after abdominal surgery. Hepatogastroenterology.

[39] De Aguilar-Nascimento JE, Marra JG, Slhessarenko N, Fontes CJF (2007). Efficacy of National Nosocomial Infection Surveillance score, acute-phase proteins, and interleukin-6 for predicting postoperative infections following major gastrointestinal surgery. Sao Paulo Med J.

[40] Albanopoulos K, Alevizos L, Natoudi M, Dardamanis D, Menenakos E, Stamou K (2013). C-reactive protein, white blood cells, and neutrophils as early predictors of postoperative complications in patients undergoing laparoscopic sleeve gastrectomy. Surg Endosc.

[41] Garcia-Granero A, Frasson M, Flor-Lorente B, Blanco F, Puga R, Carratalá A (2013). Procalcitonin and C-reactive protein as early predictors of anastomotic leak in colorectal surgery: a prospective observational study. Dis Colon Rectum.

[42] Guirao X, Juvany M, Franch G, Navinés J, Amador S, Badía JM (2013). Value of C-reactive protein in the assessment of organ-space surgical site infections after elective open and laparoscopic colorectal surgery. Surg Infect (Larchmt).

[43] Lagoutte N, Facy O, Ravoire A, Chalumeau C, Jonval L, Rat P (2012). C-reactive protein and procalcitonin for the early detection of anastomotic leakage after elective colorectal surgery: pilot study in 100 patients. J Visc Surg Elsevier Masson SAS.

[44] Oberhofer D, Juras J, Pavičić AM, Rančić Žurić I, Rumenjak V (2012). Comparison of C-reactive protein and procalcitonin as predictors of postoperative infectious complications after elective colorectal surgery. Croat Med J.

[45] Ramanathan ML, Mackay G, Platt J, Horgan PG, McMillan DC (2013). Impact of day 2 C-reactive protein on day 3 and 4 thresholds associated with infective complications following curative surgery for colorectal cancer. World J Surg.

[46] Shimizu T, Endo Y, Tabata T, Mori T, Hanasawa K, Tsuchiya M (2005). Diagnostic and predictive value of the silkworm larvae plasma test for postoperative infection following gastrointestinal surgery*. Crit Care Med.

[47] Siassi M, Riese J, Steffensen R, Meisner M, Thiel S, Hohenberger W (2005). Mannan-binding lectin and procalcitonin measurement for prediction of postoperative infection. Crit Care.

[48] Takakura Y, Hinoi T, Egi H, Shimomura M, Adachi T, Saito Y (2013). Procalcitonin as a predictive marker for surgical site infection in elective colorectal cancer surgery. Langenbecks Arch Surg.

[49] Veeramootoo D, Parameswaran R, Krishnadas R, Froeschle P, Cooper M, Berrisford RG (2009). Classification and early recognition of gastric conduit failure after minimally invasive esophagectomy. Surg Endosc.

[50] Welsch T, Frommhold K, Hinz U, Weigand MA, Kleeff J, Friess H (2008). Persisting elevation of C-reactive protein after pancreatic resections can indicate developing inflammatory complications. Surgery.

[51] Scepanovic MS, Kovacevic B, Cijan V, Antic A, Petrovic Z, Asceric R (2013). C-reactive protein as an early predictor for anastomotic leakage in elective abdominal surgery. Tech Coloproctol.

[52] Singh PP, Zeng ISL, Srinivasa S, Lemanu DP, Connolly AB, Hill A (2014). GSystematic review and meta-analysis of use of serum C-reactive protein levels to predict anastomotic leak after colorectal surgery. Br J Surg.

[53] Kubo T, Ono S, Ueno H, Shinto E, Yamamoto J, Hase K (2013). Elevated preoperative C-reactive protein levels are a risk factor for the development of postoperative infectious complications following elective colorectal surgery. Langenbecks Arch Surg.

[54] Tsujimoto H, Ono S, Takahata R, Hiraki S, Yaguchi Y, Kumano I (2012). Systemic inflammatory response syndrome as a predictor of anastomotic leakage after esophagectomy. Surg Today.

