# Supplementary material for: Diagnostic value of C-reactive protein to rule out infectious complications after major abdominal surgery: a systematic review and meta-analysis
Source: Int J Colorectal Dis. 2015 May 3;30(7):861–73. doi: 10.1007/s00384-015-2205-y (PMC4471323; doi:10.1007/s00384-015-2205-y)
Supplement: Supplementary file 1 — (124 kb) [file 384_2015_2205_MOESM1_ESM.docx]

Supplement A

1.1 MEDLINE

- Search date: January 26, 2014

|  | **MeSH Terms** | **All Fields** |
| --- | --- | --- |
|  |  |  |
| *Diagnostic modality* | | |
| CRP | "C-Reactive Protein" | (CRP) OR (C-Reactive Protein) |
| **AND** | | |
| *Population* | | |
| Abdominal surgery | "digestive system surgical procedures" | abdominal surgery[ti] |
| OR | | |
| Post-operative |  | surg*[Ti] OR resect*[ti] OR opera*[ti] |
| OR | | |
| Abdominal organs |  | (esophagect*[ti] OR gastrect*[ti] OR pancreatect*[ti] OR pancreaticoduodenect*[ti] OR pancreatoduodenect*[ti] OR hepatect*[ti] OR colect*[ti]) |

1.2 EMBASE

- Search date: January 26, 2014

|  | **Search term** | |
| --- | --- | --- |
|  |  |  |
| *Diagnostic modality* | | |
| CRP | exp C reactive protein/ | |
| **AND** | | |
| *Population* | | |
| Abdominal surgery | abdominal surgery/ | |
| OR | | |
| Post-operative | (surg* OR resect* OR opera*).m_titl. | |
| OR | | |
| Abdominal organs | (esophagect* OR gastrect* OR pancreatect* OR pancreaticoduodenect* OR pancreatoduodenect* OR hepatect* OR colect*).m_titl. | |

1.3 Cochrane

- Search date: January 26, 2014

|  | **MeSH Terms** | **All Fields** |
| --- | --- | --- |
|  |  |  |
| *Diagnostic modality* | | |
| CRP | "C-Reactive Protein" | (CRP) OR (C-Reactive Protein) |
| **AND** | | |
| *Population* | | |
| Abdominal surgery | "digestive system surgical procedures" | abdominal surgery[ti] |
| OR | | |
| Post-operative |  | surg*[Ti] OR resect*[ti] OR opera*[ti] |
| OR | | |
| Abdominal organs |  | (OR esophagect*[ti] OR gastrect*[ti] OR pancreatect*[ti] OR pancreaticoduodenect*[ti] OR pancreatoduodenect*[ti] OR hepatect*[ti] OR colect*[ti]) |

Table S1. Diagnostic accuracy of the individual studies

| **Reference** | **N** | **Incidence complications** | **POD** | **AUC** | **Cut off value^*^** | **PPV** | **NPV** | **Sens** | **Spec** | **TP** | **FN** | **FP** | **TN** |
| --- | --- | --- | --- | --- | --- | --- | --- | --- | --- | --- | --- | --- | --- |
| Aguilar-Nascimento  2007  Brasil(43) | 32 | 50%  (16/32) | NS | NS | NS | NS | NS | NS | NS | NS | NS | NS | NS |
| Albanopoulos  2012  Greece(44) | 177 | 5%  (9/177) | 0 | 0.97  (0.0-1.0) | 55 | NS | NS | 100 | 90.3 | 9 | 0 | 16 | 152 |
|  |  |  | 1 | 0.967  (0.0-1.0) | 150 | NS | NS | 83.2 | 100 | 8 | 2 | 0 | 168 |
|  |  |  | 3 | 1.0  (0.0-1.0) | 200 | NS | NS | 100 | 100 | 9 | 0 | 0 | 168 |
|  |  |  | 5 | 0.99  (0.0-1.0) | 150 | NS | NS | 100 | 98.4 | 9 | 0 | 3 | 165 |
|  |  |  | 7 | 0.97  (0.0-1.0) | 130 | NS | NS | 88.1 | 96.6 | 8 | 1 | 6 | 162 |
|  |  |  | 9 | 0.91  (0.729-1.0) | 60 | NS | NS | 85.7 | 79.6 | 8 | 1 | 34 | 134 |
|  |  |  | 11 | 0.93  (0.849-1.0) | 60 | NS | NS | 100 | 82.2 | 9 | 0 | 30 | 138 |
|  |  |  | 13 | 0.96  (0.0-1.0) | 55 | NS | NS | 100 | 86.5 | 9 | 0 | 23 | 145 |
|  |  |  | 30 | 0.88  (0.74-1.0) | 30 | NS | NS | 77.8 | 88 | 7 | 2 | 20 | 148 |
| Dutta  2011  UK(14) | 136 | 39.7%  (54/136) | 3 | 0.74  (0.06-0.85) | 180 | NS | NS | 71 | 65 |  |  |  |  |
|  |  |  | 4 | 0.79  (0.68-0.9) | 180 | NS | NS | 61 | 86 |  |  |  |  |
| Garcia-Granero  2013  Spain(45) | 205 | 22.9%  (47/205) | 3 | 0.88(0.74-1.08) | 147 | 12 | 99 | 91 | 61 | 43 | 4 | 62 | 96 |
|  |  |  | 4 | 0.81(0.74-1.08) | 101 | 11 | 99 | 91 | 60 | 43 | 4 | 63 | 95 |
|  |  |  | 5 | 0.85(0.46-0.99) | 135 | 20 | 98 | 73 | 83 | 34 | 13 | 27 | 131 |
| Guirao  2013  Spain(19) | 208 | 18.3%  (38/208) | 2 Open | 0,67  (0,567-0,76) | 240 | 40  (25,6-56,4) | 83,9  (72,8-91,0) | 58,3  (36,7-77,9) | 71,2  (59,4-81,2) | 16 | 38 | 170 | 121 |
|  |  |  | 2 Lap | 0,72  (0,62-0,81) | 186 | 36,8  (19,1-59,0) | 92,5  (84,4-96,4) | 53,8  (25,2-80,7) | 86  (76,9-92,6) | 18 | 20 | 24 | 146 |
|  |  |  | 5 Open | 0,59  (0,89-0,99) | 120 | 60  (40,7-76,6) | 100  (93,6-100) | 100  (78-100) | 84,8  (73,9-92,5) | 4 | 34 | 26 | 144 |
|  |  |  | 5 Lap | 0,92  (0,84-0,97) | 66 | 45,5  (28,8-68,8) | 98,4  (91,3-99,7) | 90,9  (58,7-98,5) | 83,3  (72,7-91,1) | 35 | 3 | 28 | 142 |
| Lagoutte  2012  France(46) | 100 | 32%  (32/100) | Pre ok | 0.72 | NS | NS | NS | NS | NS | NS | NS | NS | NS |
|  |  |  | 1 | 0.63 | NS | NS | NS | NS | NS | NS | NS | NS | NS |
|  |  |  | 2 | 0.71 | NS | NS | NS | NS | NS | NS | NS | NS | NS |
|  |  |  | 3 | 0.80 | NS | NS | NS | NS | NS | NS | NS | NS | NS |
|  |  |  | 4 | 0.87 | NS | NS | NS | NS | NS | NS | NS | NS | NS |
| Mackay  2009  UK(2) | 150 | 21.3%  (32/150) | 1 | 0.54 | 82 | 21 | 96 | 95 | 24 | 30 | 2 | 90 | 28 |
|  |  |  | 2 | 0.68 | 164 | 31 | 90 | 79 | 52 | 25 | 7 | 57 | 61 |
|  |  |  | 3 | 0.82 | 195 | 55 | 91 | 72 | 83 | 23 | 9 | 20 | 98 |
|  |  |  | 4 | 0.87 | 145 | 61 | 96 | 85 | 86 | 27 | 5 | 17 | 102 |
|  |  |  | 5 | 0.88 | 135 | 74 | 93 | 79 | 90 | 25 | 7 | 12 | 106 |
| Matsuda  2008  Japan(25) | 41 | 29.2%  (12/41) | NS | NS | NS | NS | NS | NS | NS | NS | NS | NS | NS |
| Matthiessen  2006  Sweden(1) | 33 | 27.2%  (9/33) | NS | NS | NS | NS | NS | NS | NS | NS | NS | NS | NS |
| Natsume  2011  Japan(55) | 42 | 19%  (8/42) | NS | NS | NS | NS | NS | NS | NS | NS | NS | NS | NS |
| Oberhofer  2012  Croatia(47) | 79 | 36,6%  (29/80) | 3 | 0.75  (0.63-0.84) | 99 | NS | NS | 75.9 | 68 | 22 | 7 | 16 | 34 |
|  |  |  | 5 | 0.85 | 48 | NS | NS | NS | NS | NS | NS | NS | NS |
| Ortega –Deballon  2010  France(30) | 133 | Septic complications 39,1% (52/133) | 2 | 0,71 | NS | NS | NS | NS | NS | NS | NS | NS | NS |
|  |  |  | 4 | 0,80 | NS | NS | NS | NS | NS | NS | NS | NS | NS |
|  |  |  | 6 | 0,79 | NS | NS | NS | NS | NS | NS | NS | NS | NS |
| Platt  2012  UK(13) | 454 | 23%  (104/454) | 3 | 0.80 (0.74-0.85) | 170 | NS | NS | 74 | 75 | 77 | 27 | 87 | 263 |
|  |  |  | 4 | 0.79 | NS | NS | NS | NS | NS | NS | NS | NS | NS |
| Ramanathan  2013  UK(48) | 357 | 23%  (83/357) | 2 | 0.63 | 200 | NS | NS | NS | NS | NS | NS | NS | NS |
| Reith  1998  Germany(37) | 35 | 20%  (7/35) | NS | NS | NS | NS | NS | NS | NS | NS | NS | NS | NS |
| Sarbinowski  2005  Sweden(56) | 50 | 48%  (24/50) | NS | NS | NS | NS | NS | NS | NS | NS | NS | NS | NS |
| Scepanovic  2013  Serbia and Montenegro(49) | 156 | 15.4%  (24/156) | 1 | 0.69  (0.51-0.80) | 187 | NS | NS | 53 | 70 | 13 | 11 | 40 | 92 |
|  |  |  | 2 | 0.73  (0.59-0.86) | 164 | NS | NS | 73 | 64 | 18 | 6 | 48 | 84 |
|  |  |  | 3 | 0.74  (0.59-0.89) | 135 | NS | NS | 73 | 73 | 18 | 6 | 36 | 96 |
|  |  |  | 4 | 0.75  (0.60-0.90) | 116 | NS | NS | 67 | 77 | 16 | 8 | 30 | 102 |
|  |  |  | 5 | 0.76  (0.61-0.90) | 114 | NS | NS | 67 | 90 | 16 | 8 | 13 | 119 |
|  |  |  | 6 | 0.78  (0.63-0.93) | 103 | NS | NS | 67 | 90 | 16 | 8 | 13 | 119 |
|  |  |  | 7 | 0.83  (0.69-0.97) | 85 | NS | NS | 80 | 90 | 19 | 5 | 13 | 119 |
| Shimizu  2005  Japan(50) | 112 | 34.8%  (39/112) | 7 | 0.77 | 4.6 | 61.7 | 84.6 | 74.4 | 75.3 | 29 | 10 | 18 | 55 |
| Siassi  2005  Germany(51) | 162 | 15%  (25/162) | NS | NS | NS | NS | NS | NS | NS | NS | NS | NS | NS |
| Takakura  2013  Japan (52) | 114 | 15.7%  (18/114) | 3 | 0.71 | 9.5mg/dL | 25.9 | 93.3 | 77.7 | 58.3 | 14 | 4 | 40 | 56 |
| Van Genderen  2011  The Netherlands(4) | 63 | 56%  (35/63) | 1 | 0.74(0.60-0.88) | 108.5 | NS | NS | 82 | 50 | 29 | 6 | 14 | 14 |
|  |  |  | 2 | 0.78(0.65-0.92) | 175 | NS | NS | 85 | 53 | 30 | 5 | 13 | 15 |
| Veeramootoo  2009  UK(53) | 50 | 48%  (24/50) | NS | NS | NS | NS | NS | NS | NS | NS | NS | NS | NS |
| Welsch  2007  Germany(54) | 151 | 60%  (91/151) | 3 | 0.72 | NS | NS | NS | NS | NS | NS | NS | NS | NS |
|  |  |  | 4 | 0.86 | 130 | 85.2 | NS | 78 | 74 | 71 | 20 | 16 | 44 |
|  |  |  | 5 | 0.83 | NS | NS | NS | NS | NS | NS | NS | NS | NS |
|  |  |  | 6 | 0.78 | NS | NS | NS | NS | NS | NS | NS | NS | NS |
| Welsch  2007  Germany(5) | 96 | 50%  (48/96) | 2 | 0.8 | 140 | NS | NS | 86.7 | 47.8 | 42 | 6 | 25 | 23 |
|  |  |  | 3 | 0.88 | 140 | NS | NS | 80 | 81 | 38 | 10 | 9 | 39 |
|  |  |  | 4 | 0.88 | 140 | NS | NS | 54.3 | 92.3 | 26 | 22 | 4 | 44 |
|  |  |  | 3+4 | 0.86 | 140 | NS | NS | 68.9 | 84.6 | 33 | 15 | 7 | 41 |

* Values in mg/L unless otherwise specified

POD= post-operative day, AUC=area under the receiver operator curve, PPV= positive predictive value, NPV=negative predictive value

Sens= sensitivity, Spec= specificity, TP= True Positives, FN= False Negatives, FP= False positives, TN= True Negatives

Table S2 Values of CRP comparing complicated and uncomplicated postoperative course

| **Reference** | **POD** | **Uncomplicated** | | | **Complicated** | | | **P value** |
| --- | --- | --- | --- | --- | --- | --- | --- | --- |
|  |  | **CRP** | **95% CI** | **SD** | **CRP** | **95% CI** | **SD** |  |
| Aguilar-Nascimento  2007  Brasil(43)  Mean;95%CI, mg/L | 0 | 2.0 | 0.3-3.7 |  | 3.3 | 1.5-5.1 |  | <0.001 |
|  | 2 | 20.3 | 18.1-22.6 |  | 28.7 | 26.4-31.1 |  | <0.001 |
|  | 5 | 9.6 | 14.9-23.6 |  | 19.3 | 14.9-23.6 |  | <0.001 |
| Albanopoulos  2012  Greece(44)  Mean; SD, mg/L | 0 | 30.4 |  | 18.3 | 213.0 |  | 100.1 | <0.00 |
|  | 1 | 43.7 |  | 29.2 | 208.4 |  | 141.1 | <0.00 |
|  | 3 | 67.6 |  | 33.5 | 257.2 |  | 58.7 | <0.00 |
|  | 5 | 54.9 |  | 26.6 | 242.9 |  | 60.6 | <0.00 |
|  | 7 | 41.2 |  | 23.8 | 216.8 |  | 95.4 | <0.00 |
|  | 9 | 40.2 |  | 39.8 | 161.7 |  | 134.6 | <0.00 |
|  | 11 | 41.9 |  | 20.3 | 156.7 |  | 90.4 | <0.00 |
|  | 13 | 27.8 |  | 16.8 | 122.4 |  | 49.27 | <0.00 |
|  | 30 | 14.0 |  | 7.0 | 48.2 |  | 21.7 | 0.01 |
| Dutta  2011  UK(14)  Median; range; mg/L | Pre op | 4.5 (1-77) |  |  | 3.6 (1-85) |  |  | 0.515 |
|  | 1 | 114 (10-214) |  |  | 140.5 (25-306) |  |  | 0.01 |
|  | 2 | 196.5 (53-328) |  |  | 216 (95-350) |  |  | 0.077 |
|  | 3 | 152.5 (42-198) |  |  | 183.5 (61-392) |  |  | 0.006 |
|  | 4 | 108 (37-226) |  |  | 163.5 (40-393) |  |  | <0.001 |
|  | 5 | 80.5 (18-293) |  |  | 196.5 (25-321) |  |  | <0.001 |
|  | 6 | 66 (16-312) |  |  | 181 (16-395) |  |  | <0.001 |
|  | 7 | 55 (13-293) |  |  | 179.5 (12-429) |  |  | <0.001 |
| Garcia-Granero  2013  Spain(45)  Mean; SD; mg/L | 1 | 89.4 |  | 46.8 | 137.3 |  | 51.1 | 0.001 |
|  | 2 | 150.4 |  | 66.7 | 196.0 |  | 73.1 | 0.012 |
|  | 3 | 135.2 |  | 72.6 | 192.6 |  | 77.5 | 0.002 |
|  | 4 | 102.8 |  | 68.9 | 171.8 |  | 102.5 | <0.001 |
|  | 5 | 77.1 |  | 63.2 | 177.0 |  | 102.2 | <0.001 |
| Guirao  2013  Spain(19)  Mean; 95%CI: mg/L | 2 Open | 192 | 174-210 |  | 244 | 206-281 |  | <0.01 |
|  | 2 laparoscopic | 132 | 118-146 |  | 190 | 139-242 |  | <0.01 |
|  | 5 open | 84 | 81-88 |  | 236 | 198-275 |  | <0.001 |
|  | 5 laparoscopic | 57 | 44-70 |  | 203 | 132-273 |  | <0.001 |
| Lagoutte  2012  France(46)  Median; mg/L | Pre ok | 4.7 |  |  | 23.3 |  |  |  |
|  | 1 | 124 |  |  | 155 |  |  |  |
|  | 2 | 152 |  |  | 221 |  |  |  |
|  | 3 | 118.5 |  |  | 230.5 |  |  |  |
|  | 4 | 85 |  |  | 208 |  |  |  |
| Mackay  2009  UK(2)  Median,mg/ml | 1 | 120 |  |  | 120 |  |  |  |
|  | 2 | 155 |  |  | 185 |  |  |  |
|  | 3 | 130 |  |  | 225 |  |  |  |
|  | 4 | 75 |  |  | 175 |  |  |  |
|  | 5 | 60 |  |  | 160 |  |  |  |
| Matsuda  2008  Japan(25)  Mean; mg/L | -1 | 10 |  |  | 10 |  |  |  |
|  | 1 | 80 |  |  | 100 |  |  | <0.05 |
|  | 3 | 90 |  |  | 155 |  |  | <0.05 |
|  | 5 | 45 |  |  | 90 |  |  | <0.05 |
|  | 7 | 25 |  |  | 65 |  |  | <0.05 |
| Matthiessen  2006  Sweden(1)  Median; mg/L | 1 | 170 |  |  | 180 |  |  | 0.575 |
|  | 2 | 190 |  |  | 280 |  |  | 0.004 |
|  | 3 | 130 |  |  | 275 |  |  | <0.001 |
|  | 4 | 70 |  |  | 290 |  |  | <0.001 |
|  | 5 | 50 |  |  | 285 |  |  | <0.001 |
|  | 6 | 40 |  |  | 230 |  |  | <0.001 |
|  | 7 | 40 |  |  | 200 |  |  | <0.001 |
|  | 8 | 50 |  |  | 180 |  |  | <0.001 |
| Oberhofer  2012  Croatia(47)  Median;mg/L | Pre op | 10 |  |  | 10 |  |  |  |
|  | 1 | 80 |  |  | 90 |  |  | 0.026 |
|  | 2 | 130 |  |  | 160 |  |  | 0.002 |
|  | 3 | 90 |  |  | 140 |  |  | <0.001 |
|  | 5 | 40 |  |  | 100 |  |  | <0.001 |
| Ortega –Deballon  2010  France(30)  Mean; mg/L | 2 | 155,9 |  |  | 212 |  |  |  |
|  | 4 | 83,4 |  |  | 167,3 |  |  |  |
|  | 6 | 50,5 |  |  | 120,7 |  |  |  |
| Platt  2012  UK(13)  Median; range, mg/L | Pre op | 8 (1-222) |  |  | 14 (1-317) |  |  | <0.001 |
|  | 1 | 108 (5-348 ) |  |  | 125 (14-343) |  |  | 0.064 |
|  | 2 | 163 (17-356) |  |  | 215 (82-358) |  |  | <0.001 |
|  | 3 | 132 (6-319) |  |  | 208 (38-352) |  |  | 0.001 |
|  | 4 | 90 (6-306) |  |  | 149 (23-317) |  |  | <0.001 |
|  | 5 | 59 (5-265) |  |  | 108 (17-283) |  |  | <0.001 |
|  | 6 | 47 (5-28 ) |  |  | 103 (13-354) |  |  | <0.001 |
|  | 7 | 38 (5-347) |  |  | 105 (6-329) |  |  | <0.001 |
| Reith  1998  Germany(37)  Median; mg/L | Pre op | 4 |  |  | 8 |  |  |  |
|  | 1 | 5 |  |  | 16 |  |  |  |
|  | 2 | 12 |  |  | 22 |  |  |  |
|  | 3 | 14 |  |  | 20 |  |  |  |
|  | 4 | 12 |  |  | 15 |  |  |  |
|  | 5 | 6 |  |  | 16 |  |  |  |
|  | 7 | 7 |  |  | 13 |  |  |  |
|  | 10 | 4 |  |  | 11 |  |  |  |
| Scepanovic  2013  Serbia and Montenegro(49)  Median;range;mg/L | Pre op | 9 (1-88) |  |  | 8 (1-186) |  |  |  |
|  | 1 | 188 (111-288) |  |  | 154 (30-290) |  |  |  |
|  | 2 | 182 (84-287) |  |  | 138 (30-280) |  |  |  |
|  | 3 | 168 (71-256) |  |  | 111 (20-231) |  |  |  |
|  | 4 | 144 (41-203) |  |  | 78 (15-224) |  |  |  |
|  | 5 | 121 (32-170) |  |  | 56 (11-168) |  |  |  |
|  | 6 | 115 (22-180) |  |  | 47 (9-118) |  |  |  |
|  | 7 | 122 (14-386) |  |  | 33 (3-13) |  |  |  |
| Shimizu  2005  Japan(50)  Mean;mg/L | Pre op | 20 |  |  | 40 |  |  |  |
|  | 1 | 60 |  |  | 60 |  |  | <0.05 |
|  | 3 | 100 |  |  | 160 |  |  | <0.05 |
|  | 7 | 40 |  |  | 80 |  |  | <0.05 |
| Takakura  2013  Japan (52)  NS;mg/L | 1 | 60 |  |  | 80 |  |  | 0.03 |
|  | 3 | 70 |  |  | 140 |  |  | <0.01 |
| Van Genderen  2011  The Netherlands(4)  Mean; mg/L | 0 | 0 |  |  | 0 |  |  |  |
|  | 1 | 110 |  |  | 160 |  |  |  |
|  | 2 | 180 |  |  | 220 |  |  |  |
|  | 3 | 190 |  |  | 210 |  |  |  |
| Welsch  2007  Germany(54)  Median, mg/L | Pre op | 10 |  |  | 10 |  |  |  |
|  | 1 | 60 |  |  | 70 |  |  |  |
|  | 2 | 130 |  |  | 160 |  |  |  |
|  | 3 | 135 |  |  | 170 |  |  |  |
|  | 4 | 70 |  |  | 160 |  |  |  |
|  | 5 | 65 |  |  | 150 |  |  |  |
|  | 6 | 60 |  |  | 130 |  |  |  |
|  | 7 | 40 |  |  | 135 |  |  |  |
|  | 8 | 35 |  |  | 140 |  |  |  |
|  | 9 | 40 |  |  | 150 |  |  |  |
|  | 10 | 20 |  |  | 160 |  |  |  |
|  | 11 | 60 |  |  | 165 |  |  |  |
|  | 12 | 20 |  |  | 160 |  |  |  |
| Welsch  2007  Germany(5)  Median, mg/L | Pre op | 10 |  |  | 10 |  |  |  |
|  | 1 | 90 |  |  | 130 |  |  |  |
|  | 2 | 140 |  |  | 160 |  |  |  |
|  | 3 | 110 |  |  | 150 |  |  |  |
|  | 4 | 70 |  |  | 140 |  |  |  |
|  | 5 | 60 |  |  | 150 |  |  |  |
|  | 6 | 40 |  |  | 130 |  |  |  |
|  | 7 | 30 |  |  | 130 |  |  |  |
|  | 8 | 40 |  |  | 140 |  |  |  |
|  | 9 | 45 |  |  | 150 |  |  |  |
|  | 10 | 30 |  |  | 130 |  |  |  |
|  | 11 | 60 |  |  | 125 |  |  |  |
|  | 12 | 30 |  |  | 100 |  |  |  |
